# Pharmacological Enhancement of Integrated Stress Response Confers Protection in Calcific Aortic Valve Disease

**DOI:** 10.1016/j.jacbts.2025.101433

**Published:** 2025-12-15

**Authors:** Libo Wang, Xulei Duan, Huibing Liu, Fei Lin, Chaoyuan Zhou, Katrin Schröder, Ajay M. Shah, Guoan Zhao, Min Zhang

**Affiliations:** a Department of Cardiology, Life Science Research Center, The First Affiliated Hospital of Xinxiang Medical University, Xinxiang, Henan, China; b Institute for Cardiovascular Physiology, Goethe-University, Frankfurt, Germany; c King’s College London British Heart Foundation Centre of Research Excellence, School of Cardiovascular and Metabolic Medicine & Sciences, London, United Kingdom

**Keywords:** aortic valve, apoptosis, ATF4, calcification, Nox4, reactive oxygen species

## Abstract

Previous studies have shown that Nox4 activates eIF2α/ATF4 signaling during the integrated stress response (ISR) and protects heart injury. However, their roles in calcific aortic valve disease (CAVD) remain unclear. Here, we show that both ATF4 and Nox4 are up-regulated in porcine aortic valve interstitial cells (AVIC) and in human aortic valves with CAVD. Nox4 knockdown promotes while Nox4 overexpression suppresses CAVD by modulating ISR. Importantly, ISR activators Guanabenz and Sephin1 effectively attenuate AVIC osteoblastic-like differentiation and mitigate CAVD in rabbits and mice, respectively. These findings highlight that pharmacological enhancement of the ISR is a promising therapeutic strategy for CAVD.

## Highlights

• The ISR effector ATF4 and the ROS-generating enzyme Nox4 are up-regulated in the aortic valve leaflets of mice, rabbits, and patients with CAVD.
• Knockdown of endogenous Nox4 significantly promoted calcification in cultured porcine AVIC and increased aortic valve calcium deposits in mice.
• Nox4 overexpression suppressed AVIC calcification by enhancing ISR activation through the p-eIF2α/ATF4 pathway.
• Guanabenz and Sephin1, ISR activators that prolong eIF2α phosphorylation, effectively alleviated AVIC osteoblastic-like differentiation and mitigated the severity of aortic valve calcification and stenosis in respective rabbit and mouse CAVD models.
• This study suggests that pharmacological enhancement of the adaptive integrated stress response holds therapeutic potential for targeting CAVD.

Calcific aortic valve (AV) disease (CAVD) is the most predominant form of heart valvular disorder affecting millions worldwide, and its prevalence has more than doubled since 1990 due to aging and population growth.^1^^,^^2^ CAVD is a slowly progressive disease characterized by thickening, stiffness, fibrosis, and calcification of leaflets, resulting in valve sclerosis and aortic stenosis. CAVD is associated with substantial morbidity and mortality, but there is currently no effective pharmacological therapy to prevent or alter the course of CAVD other than surgical AV replacement, or more recently, transcatheter AV implantation in select patients experiencing symptomatic severe aortic stenosis. Therefore, a major unmet clinical need exists to identify new therapeutic strategies mitigating the development of CAVD.^3^

It has been widely recognized that CAVD is an active and cellular-driven multistage disease with sequential yet intertangled processes.^4^ Crucially, activation of aortic valvular interstitial cells (AVIC), which represent the predominant cellular constituents within AVs, and their transdifferentiation into osteoblastic-like cells appears to be a central step in the disease progression,^5^ thus can be pharmacologically targeted.^6^ Numerous pathological stimuli, including mechanical stress, inflammation, reactive oxygen species (ROS) formation, lipoprotein oxidation, and altered calcium-phosphorus homeostasis, induce AVIC apoptosis and the resultant release of apoptotic bodies, which facilitate the formation of additional nidi for further calcium deposition causing an exponential increase in the rate of calcification.^7^^,^^8^ Perpetuating this process, AVIC progressively acquire osteoblast-like phenotypes and characteristics with abnormal up-regulation of typical bone genes such as osteopontin (OPN) and runt-related transcription factor 2 (Runx2), which are critically involved in osteogenesis and ectopic mineralization.^9^^,^^10^ However, the exact pathophysiology underlying the initial loss of AVIC and progression of CAVD remain not fully understood. Better understanding the cellular and molecular mechanisms especially related to AVIC protection will open novel avenues for the effective treatment of this debilitating disease.

The integrated stress response (ISR) is an evolutionarily conserved cytoprotective signaling pathways that maintains cellular proteostasis.^11^ The ISR is triggered by diverse cytotoxic insults, including redox imbalance, lipid deposition, and endoplasmic reticulum (ER) stress, all of which have been implicated in CAVD pathogenesis.^7^^,^^12^^,^^13^ The focal point of ISR activation is the phosphorylation of the alpha subunit of the eukaryotic initiation factor 2 (eIF2α) at Ser51, leading to an overall attenuation of protein synthesis, concurrent with preferential translation of specific proteins such as activating transcriptional factor 4 (ATF4), which functions in adaptation to stress and promotes cellular recovery.^14^ Once cellular hemostasis is re-established, protein synthesis is resumed via a negative feedback control protein, in which ATF4 induces expression of C/EBP-homologous protein (CHOP), which in turn promotes expression of growth arrest and DNA damage-inducible 34 (GADD34). GADD34 binds to protein phosphatase 1 catalytic subunit (PP1c) to dephosphorylate eIF2α, ensuring reinitiation of protein synthesis.^11^ If cellular stress cannot be mitigated, however, the accumulation of CHOP leads to apoptosis.^15^

It is now clear that ISR is centrally implicated in cellular stress adaptation and fate-decision, growing evidence supports that dysregulation of ISR signaling critically contributes to the pathogenesis of diverse disorders including neurodegenerative,^16^ tumoral,^17^ respiratory,^18^ metabolic, and cardiovascular diseases.^19^^,^^20^ In this light, extensive studies have been conducted to elucidate the mechanisms underlying the modulation of ISR, with an aim to identify specific compounds to pharmacologically tune the activity of ISR to address pathologies. Guanabenz (2,6-dichlorobenzylidene aminoguanidine acetate [Gbz]), which is a classic antihypertensive drug as an α2-adrenergic receptor agonist approved by the U.S. Food and Drug Administration, was reported to grant protection in an experimental autoimmune encephalomyelitis mouse model by inhibiting the binding of GADD34 to PP1c, thereby maintaining the phosphorylation of eIF2α.^21^^,^^22^ Later on, a Guanabenz derivative, Sephin1, which selectively and specifically inhibits GADD34-PP1c binding without measurable α2-adrenergic side effects,^23^ has also been shown to ameliorate the progression of multiple sclerosis.^24^^,^^25^ Moreover, an intriguing mechanism by which ISR can be modulated is through a targeted oxidative inactivation of the PP1. Previous work in the authors’ laboratory revealed that NADPH oxidase-4 (Nox4), a ROS-generating protein that is induced by ATF4, can bind GADD34 and inhibit PP1 activity to sustain eIF2a phosphorylation and increase ATF4 expression.^26^ Importantly, either elevation of Nox4 levels or Gbz treatment exhibits beneficial cardiac and renal effects through the improvement of cell survival under stress.^26^ These studies suggest that the enhancement of ISR signaling by small-molecule inhibitors of the phosphatase complexes or activation of Nox4-mediated pathways may be a promising therapeutic approach in multiple disease settings. The involvement and contribution of Nox4, however, and the effectiveness of ISR activators on the development of CAVD are currently unknown.

Here, we report that Nox4 involves valvular adaptation to calcification through the up-regulation of cellular ISR in AVIC. Gbz treatment, which prolongs p-eIF2α/ATF4 activation, significantly diminishes the apoptosis and osteoblastic-like transdifferentiation of AVIC in vitro, as well as AV calcification and stenosis in a rabbit CAVD model in vivo. This beneficial effect of ISR enhancer is further affirmed with the use of Sephin1, which effectively improves AVIC survival and dampens the severity of AV calcification in a mouse CAVD model. Our studies provide the first evidence that pharmacological enhancement of adaptive stress response has encouraging therapeutic potential for the treatment of CAVD, as well as uncovering a previously unrecognized role of Nox4 in AV calcification.

## Methods

### Human AV tissues

Human AVs were collected from patients who underwent AV replacement surgery.^27^ Two types of AVs were obtained: control noncalcified AVs with aortic dissection and calcified AVs. Exclusion criteria included cases with bicuspid AVs, moderate-to-severe AV regurgitation, infective endocarditis, congenital valve disease, and rheumatic AV disease. Upon retrieval, the AV samples were separated into different parts. One part was fixed in 4% paraformaldehyde for histological analysis, while the remaining portions were rapidly frozen in liquid nitrogen and stored at −80 °C for future use. The study design adhered to the principles outlined in the Declaration of Helsinki, and approved by the Ethics Review Committee of the First Affiliated Hospital of Xinxiang Medical University.

### Animal studies

All animal experiments were conducted in accordance with the requirements of the National Act on the use of experimental animals in China and the Directive 2010/63/EU of the European Parliament on the protection of animals used for scientific purposes. The study protocols were approved by the Ethics Committee of The First Affiliated Hospital of Xinxiang Medical University, the University Animal Care Committee and the Federal Authorities for Animal Research in Darmstadt (Germany).

### Rabbit CAVD model

To investigate the therapeutic effects of Guanabenz, we conducted the study using a well-established rabbit model of CAVD as previously reported.^27^ Briefly, male New Zealand white rabbits (weight, 2.0-2.5 kg) were randomly assigned into 3 groups: 1) Control group (Con)— rabbits were fed a normal chow diet without any dietary supplement; 2) CAVD group (CAVD)—rabbits were fed a diet enriched with 0.5% cholesterol (WAKO Inc) and received a daily supplement of 25,000 IU of vitamin D2 (Sigma) in their drinking water; and 3) Guanabenz treatment group (CAVD+Gbz)—rabbits fed high-cholesterol (HC) diet and vitamin D supplements were orally administered Guanabenz (HuBei XinKang Pharmaceutical Chemical Co, Ltd, 23256-50-0, 99% purity) at a daily dose of 1 mg/kg. Throughout the 18-week study period, the rabbits were individually housed in cages under standard housing conditions and provided with food ad libitum. At the conclusion of the experiment, the rabbits were euthanized, and AV tissue samples were collected for further analysis.

### Rabbit echocardiography

Rabbit AV function were assessed by echocardiography using a Vivid E9 ultrasound system with a 2.5-10.0 MHz phased-array transducer (GE Healthcare).^27^ Both parasternal long-axis view and parasternal short-axis view were used to observe leaflet morphology and the opening of AVs. Aortic valve area (AVA) and flow velocity were measured and calculated following previously established methods.^27^^,^^28^ Echocardiographic imaging and analyses were performed by an experienced operator blinded to the assignments.

### Mouse CAVD model

To explore the potential effects of Sephin1, we utilized a previously reported adenine-vitamin D-induced mouse model of AV calcification with minor modifications.^29^ Male C57BL/6J mice aged 10 to 12 weeks were housed in a pathogen-free facility under controlled conditions: temperature 20 to 26 °C, humidity 40% to 70%, and a 12-hour light/dark cycle. Mice were randomly assigned to 3 groups: 1) Control group (Con)—mice fed a normal diet; 2) CAVD group (CAVD)—mice fed an adenine diet containing 1.48 × 10^−2^ mol/kg of adenine (A8626; Sigma) and 10% casein (C110500; Aladdin) for 3 weeks, followed by intraperitoneal administration of vitamin D (2.27 × 10^−5^ mol/kg body weight; C9756, Sigma) for 10 consecutive days; and 3) Sephin1 treatment group (CAVD+sephin1)—adenine-vitamin-D–treated mice received intraperitoneal injections of sephin1 (1 mg/kg, once a day, IFB-080, MedChemExpress) for the last 10 days. At the end of the treatment period, Doppler ultrasound and B-mode echocardiography were performed using high-resolution ultrasound (40 MHz, Vevo3100, VisualSonics) to evaluate the AV function. Subsequently, the mice were sacrificed, and their hearts with aortic roots were carefully dissected and embedded for further analyses, including Alizarin Red staining and immunofluorescence.

### Nox4 knockout mice

To investigate the involvement of endogenous Nox4 in the development of CAVD, we employed a tamoxifen-inducible Nox4 knockout mouse model (Nox4^flox/flox^-C57/Bl6J with Cre-ERT2-transgenics), which was bred in the ApoE^−/−^ background. Animals were bred under standard conditions with 12/12-hour dark/light cycle. Activation of Cre-ERT2 was achieved at an animal age of 7 weeks. Following tamoxifen treatment, the mice were fed a standard normal chow diet and allowed to age for 9 months.^30^ After the designated period, mouse AVs were harvested and stained to visualize and quantify calcium deposition, expressed as the percentage of positively stained area. Only male mice were used in this study.

### Histology

Aortic valves were excised, fixed in 4% paraformaldehyde, paraffin-embedded, and then sectioned (5 μm). Calcium deposits were assessed by Alizarin Red staining. Additionally, hydroxyapatite (HAP), a well-established marker of calcification, was detected using the fluorescent probe OsteoSense 680 (IVISense, Revvity). Fluorescence intensity was quantified in ImageJ, and integrated density was normalized to the total valve area. To analyze the expression levels of Nox4, ATF4, and CHOP in rabbit AVs, immunostaining was carried out using specific antibodies (ab109225, Abcam; ab216839, Abcam; ab11419, Abcam) against each target. To analyze the expression levels of Nox4 and ATF4 (ab109225, Abcam; 60035-1-Ig, Proteintech) in mouse AVs, immunofluorescence staining was performed using corresponding antibodies for each target. The fractional areas were analyzed and quantified using the ImageJ software. Data are expressed as the percentage of valve area that displays positive staining.

### Cell culture and AVIC calcification

Primary porcine AVICs were isolated and cultured as described previously.^27^ Briefly, fresh pig AVs were harvested from adult domestic male pigs. AVIC were isolated by collagenase II digestion and cultured in Dulbecco’s modified Eagle medium (DMEM) (Gibco) containing 10% fetal bovine serum (Gibco), 1% penicillin and streptomycin (Gibco, Life Technologies). To induce calcification, cells were incubated with osteogenic medium (OGM) containing 50 mg/L ascorbic acid, 2 mmol/L β-glycerophosphate sodium, and 100 nmol/L dexamethasone up to 14 days. For some experiments, pharmacological reagent Guanabenz (G110, Sigma) or Sephin1 (IFB-080, MedChemExpress) was added at the final concentration of 5 μmol/L and 0.5 μmol/L, respectively. For some experiments, pan-caspase inhibitor Z-VAD(OMe)-FMK (KM11752, KKL Med) (referred to as FMK) was added at the final concentration of 2 μmol/L. All experiments were performed with cells from passages 3 to 5.

### Quantification of Calcium content

To quantify calcium content in vitro, a calcium colorimetric assay kit (S1063S, Beyotime Biotechnology) was used according to manufacturer’s instructions. Briefly, AVIC were cultured in 6-well plates, rinsed by phosphate-buffered saline (without calcium and magnesium) 3 times, and lysed in lysis buffer. A working solution (200 μL) was added to each well and incubated for 5 minutes to ensure complete cell lysis. The lysates were collected and centrifuged at 12,000 × *g* for 5 min to obtain the supernatant. Absorbance at 575 nm was measured using a Spectra max 384 plus microplate reader (Molecular Devices). A standard curve was generated by a calcium standard solution, and calcium concentrations in the samples were calculated based on their respective absorbance values.

### Adenoviral vectors

Adenoviral vectors were created to manipulate Nox4 or ATF4 expressions in AVIC. To overexpress Nox4, an adenoviral vector carrying the Nox4 gene (Gene ID:100523323) was used (referred to as ad-Nox4), with a β-galactosidase-expressing (β-gal) serving as the control vector. To knockdown Nox4 expression, a separate adenoviral vector carrying a short hairpin RNA (shRNA) sequence targeted against porcine Nox4 (GGTGCTATTCCTGATGATTACAGCTTCAA) was generated (referred to as shNox4), and a green fluorescent protein (GFP)-expressing vector was used as the control vector. To knockdown ATF4 expression in AVIC, an adenoviral vector carrying a shRNA targeting porcine ATF4 (sequence: GGAAATCTCGGAAGGAGATAG) was constructed (referred to as shATF4). AVIC were infected with respective adenoviral vectors at a multiplicity of infection of 10 for 24 hours. Subsequently, the cells were washed with phosphate-buffered saline before being exposed to either normal medium or OGM.

### Alizarin Red staining

Calcium deposition was examined using filtered 2% Alizarin Red solution (A5533, Sigma). Valve tissues or cells were fixed with 4% paraformaldehyde for 15 minutes at room temperature and excessive dye was removed by washing with distilled water. For the staining of cells with Alizarin Red, use Alizarin Red staining solution for 15 minutes, remove the staining solution. For valve tissue slices, paraffin sections were stained by alizarin red solution for 5 minutes after the consecutive procedures of deparaffinized and rehydrated, then wash 3 times with distilled water.

### Western blotting

Cell lysates or AV homogenates were subjected to Western blot analysis as described previously.^27^ The primary antibodies used were: Nox4 (ab109225, Abcam); Runx2 (8486, Cell Signaling); Osteopontin (ab8448, Abcam); p-eIF2α (ab32157, Abcam); eIF2α (11170-1-AP, Proteintech); ATF4 (ab1371, Abcam); KDEL (ab69659, Abcam); Cleaved caspase-12 (2202, Cell Signaling); Caspase-12 (55238-1-AP, Proteintech); CHOP (ab11419, Abcam); GAPDH (10494-1-AP, Proteintech). GPADH was used as a loading control protein. Blots were visualized using an enhanced chemiluminescence detection system ABI800, and quantified by densitometry using the ImageJ software.

### Statistics

Data are presented using actual data points and the mean ± SEM. Student's unpaired *t*-test or Mann–Whitney test was utilized to compare two groups, while 1-way or 2-way analysis of variance with Tukey’s post hoc test was used for comparisons among >2 groups. The Shapiro-Wilk test was used to test the normality of the data distribution. Kaplan-Meier curves were used to evaluate mortality rates among groups. Analyses were performed on GraphPad Prism 9.0.0 for Windows (GraphPad Software).^27^ A *P* value <0.05 was considered statistically significant.

## Results

### Up-regulation of Nox4 and ATF4 in human AVs with CAVD and during AVIC calcification

**Figure 1:**
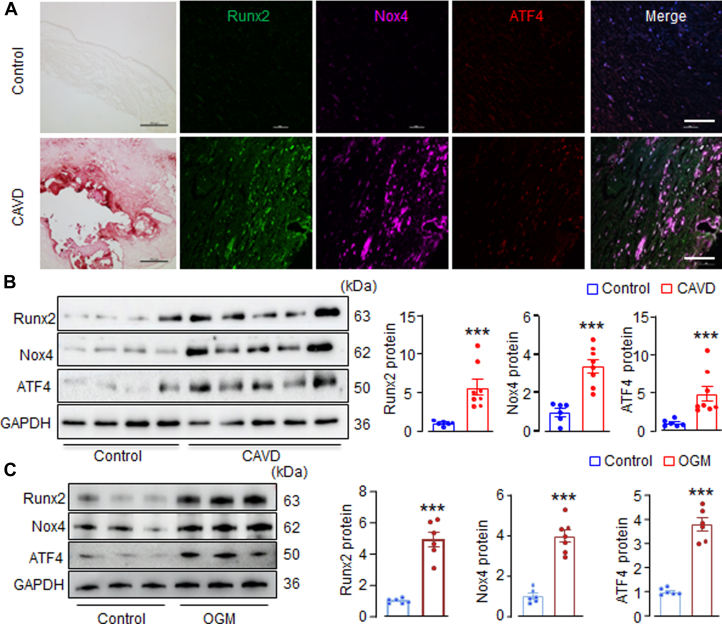
Up-Regulation of Nox4 and ATF4 in Human Aortic Valve Tissues With CAVD and During AVIC Calcification (A) Alizarin Red staining for calcification and immunofluorescence for Runx2, Nox4, and ATF4 expression in human aortic valve tissues with or without calcified aortic valve disease (CAVD). Black scale bars: 500 μm; and white scale bars: 200 μm. (B) Immunoblots of calcification marker runt-related transcription factor 2 (Runx2), reduced nicotinamide adenine dinucleotide phosphate oxidase 4 (Nox4), and activating transcription factor 4 (ATF4) proteins in human aortic valve tissues with or without CAVD. GAPDH as loading control protein. Mean data at the right. n = 6-8/group. ∗∗∗*P <* 0.001, compared with noncalcific valves. (C) Western blots showing changes of protein levels of Runx2, Nox4, and ATF4 in cultured porcine aortic valvular interstitial cells (AVIC) treated with normal DMEM medium (Control) or osteogenic medium (OGM). Mean data at the right. n = 6/group. ∗∗∗*P <* 0.001 compared with control cells. Student’s unpaired *t*-test. All data are mean ± SEM.

To explore the involvement of Nox4 in CAVD, we first examined its expression in human AVs affected by CAVD. As anticipated, the diseased valves displayed substantial calcium deposition (Figure 1A) and increased levels of the calcification marker Runx2 (Figure 1B). Immunofluorescence analysis revealed that Nox4 expression was relatively low in normal valve tissues (Figure 1A). However, in CAVD valves, there was intense Nox4 staining (Figure 1A). Quantitative immunoblotting showed that Nox4 protein levels were elevated approximately 3.5-fold in CAVD valves compared with noncalcified ones (Figures 1B).

Interestingly, ATF4, a key effector of the ISR, showed a similar pattern of up-regulation. ATF4 was significantly elevated in calcified human valves, whereas it was barely detectable in normal AV tissues (Figures 1A and 1B). Both Nox4 and ATF4 were markedly increased throughout the diseased valves, where AVIC represent the predominant cellular constituents. Given that AVIC undergo a phenotypic transition from fibroblast-like to osteoblast-like cells, which plays a central role in CAVD progression, we next examined the expression profiles of Nox4 and ATF4 during AVIC calcification in vitro.

Porcine AVIC were isolated and cultured in OGM to induce osteoblastic differentiation. Runx2 proteins, a surrogate marker of AVIC calcification severity, were assessed by Western blotting. As expected, OGM treatment resulted in a significant increase in Runx2 levels compared with control cells cultured in DMEM (Figure 1C). Strikingly, Nox4 protein levels were significantly elevated after 7 days of OGM stimulation (Figure 1C), with ATF4 showing a similar up-regulation pattern. These findings suggest that Nox4 and ATF4 are up-regulated during the process of osteoblastic-like transdifferentiation and calcification of AVIC, likely functioning interactively as part of the cellular stress response.

### Nox4 protects AVIC calcification in vitro and CAVD in vivo

**Figure 2:**
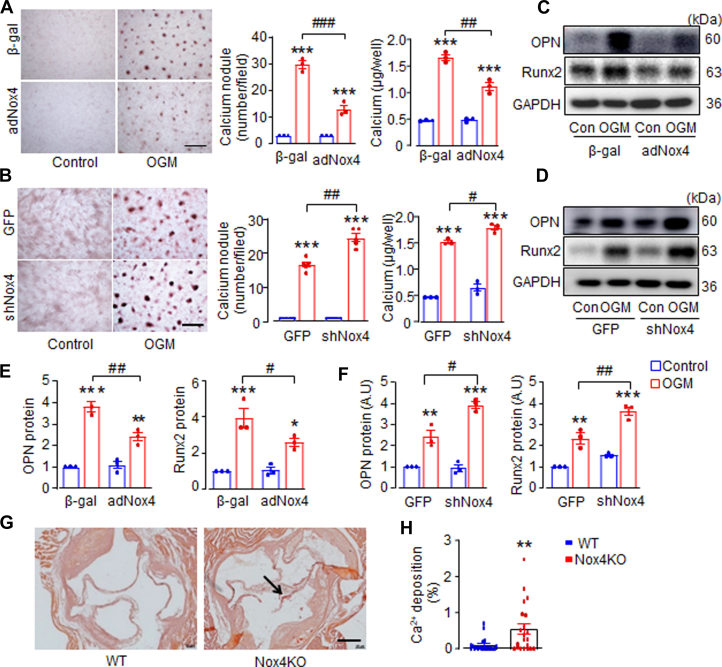
Nox4 Protects AVIC Calcification in Vitro and CAVD in Vivo (A) The effects of overexpression of Nox4 with adNox4 adenovirus, or (B) knockdown of Nox4 with shNox4 adenovirus on calcium deposition and calcium content in cultured AVIC. AVIC transfected with β-galactosidase (β-gal) virus or green fluorescent protein (GFP) virus as respective control cells. Scale bar: 100 μm. Mean data shown at the right. n = 3-5/group. (C and D) Immunoblots and (E and F) relative quantification for osteopontin (OPN) and runt-related transcription factor 2 (Runx2) in AVIC. n = 3/group. ∗*P <* 0.05, ∗∗*P <* 0.01, ∗∗∗*P <* 0.001, compared with respective control cells (Con). #*P <* 0.05, ##*P <* 0.01, ###*P <* 0.001, compared with OGM transfected with β-gal or GFP virus. Two-way analysis of variance with a post hoc Tukey’s test. (G) Representative images of Alizarin Red staining of calcium deposition and quantification (H) in aortic valves of Nox4 knockout (Nox4KO) mice compared with wild-type (WT) littermate control mice. ∗∗*P <* 0.01, compared with WT groups, Mann-Whitney test. Scale bar: 50 μm. n = 20-22/group. All data are mean ± SEM. Abbreviations as in Figure 1.

To elucidate the intrinsic role of Nox4 in AVIC calcification, both gain-of-function and loss-of-function approaches were employed to manipulate endogenous Nox4 levels. Nox4 overexpression significantly reduced calcium deposition by 56%, lowered calcium concentration by 37%, and decreased OPN and Runx2 protein levels following OGM stimulation (Figures 2A, 2C, and 2E), demonstrating the protective role of Nox4 against AVIC mineralization. Interestingly, moderate overexpression of Nox4 (∼2.8-fold) (Supplemental Figure 1A) had no effect on AVIC phenotype under normal culture condition for 2 weeks (Figures 2A, 2C, and 2E), suggesting that Nox4’s regulatory effects are specific to calcification processes. Conversely, adenoviral transfection with shNox4 significantly down-regulated Nox4 protein levels compared with the GFP virus control levels (Supplemental Figure 1B). Importantly, Nox4 knockdown resulted in a 47% increase in calcium nodule formation as shown by Alizarin Red staining, elevated calcium concentration (Figure 2B), and increased expression of calcification markers OPN and Runx2 (Figures 2D and 2F).

To further evaluate the role of endogenous Nox4 in the development of CAVD in vivo, we used a global tamoxifen-inducible Nox4 knockout (Nox4KO) mouse model bred on an ApoE^−/−^ background, as previously described.^30^ Compared with the wild-type control mice, 9-month-old Nox4KO mice exhibited significantly more calcium deposition in AVs, as evidenced by Alizarin Red staining (Figures 2G and 2H), indicating that Nox4 plays a protective role against CAVD development.

### Nox4 improves AVIC survival during calcification via activation of elF2α/ATF4 stress signaling

Next, we investigated the potential mechanism underlying the protective effect of Nox4 against CAVD. Given that Nox4 and ATF4 were both up-regulated during AVIC calcification, and ATF4 plays a critical role in ISR, we therefore explored the impact of Nox4 modulation on ISR signaling in AVIC and its functional consequences.

**Figure 3:**
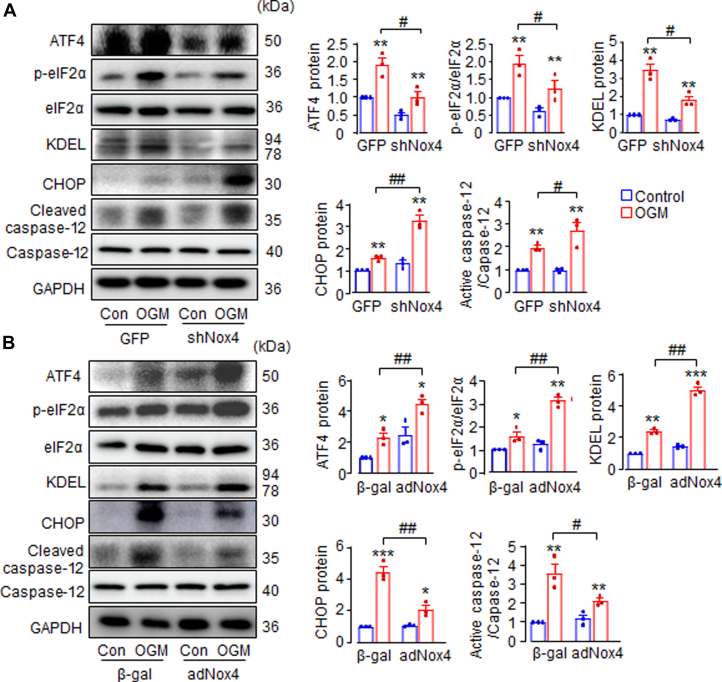
Nox4 Improves AVIC Survival During Calcification via Activation of elF2α/ATF4 Signaling (A) The effects of down-regulation of endogenous Nox4 with shNox4 adenovirus, or (B) overexpression of Nox4 with adNox4 adenovirus on protein levels of integrated stress response (ISR) markers elF2α phosphorylation and ATF4, ER chaperone KDEL, and cell survival markers CHOP and cleaved caspase-12 in cultured AVIC with or without OGM treatment. AVIC transfected with green fluorescent protein (GFP) virus or β-galactosidase (β-gal) virus as respective control cells. Mean data shown at the right. n = 3/group. ∗*P <* 0.05, ∗∗*P <* 0.01, ∗∗∗*P <* 0.001, compared with respective control cells (Con), #*P <* 0.05, ##*P <* 0.01, compared with OGM-stimulated AVIC transfected with GFP or β-gal virus. 2-way analysis of variance with a post hoc Tukey’s test. All data are mean ± SEM. CHOP = C/EBP homologous protein; elF2α = eukaryotic Initiation Factor 2; KDEL = peptide sequence Lys-Asp-Glu-Leu; other abbreviations as in Figure 1.

Indeed, ISR was activated in AVIC treated with OGM, as indicated by significantly elevated levels of eIF2α phosphorylation and ATF4 protein (Figure 3A). Additionally, OGM-treated AVIC showed increased expression of the proapoptotic markers CHOP and cleaved caspase-12, along with elevated levels of the ER stress chaperone KDEL (Figure 3A), suggesting enhanced ER stress and associated cell death.

Remarkably, Nox4 knockdown via shNox4 virus significantly suppressed OGM-induced eIF2α/ATF4 stress signaling compared with control cells transfected with GFP virus. This suppression was accompanied by higher expression of apoptotic markers CHOP and cleaved caspase-12, indicating increased cell death (Figure 3A). Conversely, Nox4 overexpression further amplified ISR activation, as evidenced by even greater levels of p-eIF2α, ATF4, and KDEL in OGM-treated AVIC. Importantly, despite the enhanced ISR, Nox4 overexpression significantly reduced cell death, as indicated by decreased levels of CHOP and the active form of caspase-12 (Figure 3B). To further investigate the role of apoptosis in Nox4-mediated AVIC calcification, the pan-caspase inhibitor FMK was applied. FMK treatment significantly reduced Nox4 knockdown-induced increase in calcium concentration by 50% (Supplemental Figure 2A) and decreased OPN and Runx2 protein levels following OGM stimulation (Supplemental Figure 2B), underscoring the critical contribution of apoptosis to Nox4-regulated AVIC mineralization.

These findings suggest that Nox4 up-regulation is part of a compensatory and adaptive response during AVIC calcification. Nox4 exerts a protective effect by enhancing AVIC survival through the activation of eIF2α/ATF4 stress signaling, thereby mitigating AVIC calcification.

### Guanabenz attenuates AVIC calcification via activation of the elF2α/ATF4 pathway

We investigated whether pharmacological activation of the eIF2α/ATF4 pathway could protect against AVIC calcification. Guanabenz (Gbz), originally used as an antihypertensive drug due to its action as an α2-adrenergic receptor agonist, has been shown to effectively activate the ISR by prolonging eIF2α phosphorylation through inhibition of the GADD34-PPIc interaction.^21^

**Figure 4:**
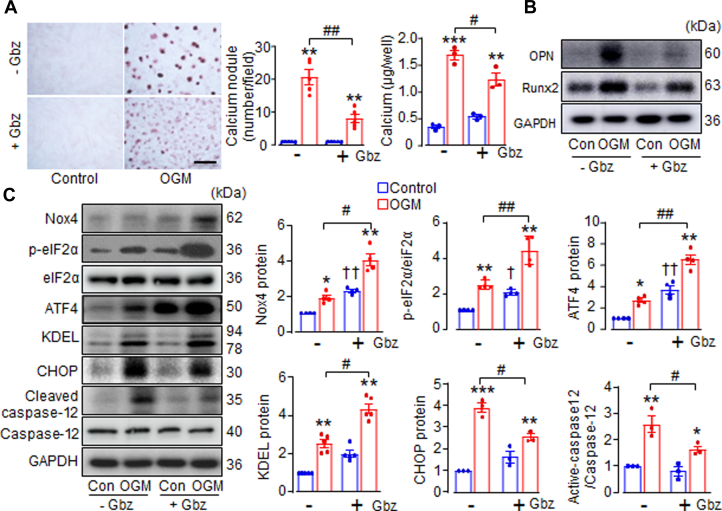
Guanabenz Attenuates AVIC Calcification Through Activation of elF2α/ATF4 Pathway (A) Calcium deposition by Alizarin Red staining and calcium concentration with or without Guanabenz (Gbz, 5 μmol/l, 14 days) treatment. Scale bar: 100 μm. Mean data shown at the right. (B) Immunoblots of calcification markers OPN and Runx2. (C) Western blots showing the effects of Gbz on expression of Nox4, p-eIF2α/ATF4 stress signaling, KDEL, and survival markers CHOP and cleaved caspase-12 in AVIC. Mean data shown at the right. n = 3-5/group, ∗*P <* 0.05, ∗∗*P <* 0.01, ∗∗∗*P <* 0.001, compared with respective control cells (Con), †*P <* 0.05, ††*P <* 0.01, compared with control cells without Gbz treatment, #*P <* 0.05, ##*P <* 0.01, compared with OGM without Gbz treatment. Two-way analysis of variance with a post hoc Tukey’s test. All data are mean ± SEM. Abbreviations as in Figure 1, Figure 2, Figure 3.

Incubation of AVIC with 5 μmol/L Gbz for 14 days had no significant effect on basal mineralization (Figures 4A and 4B), though it did mildly increase eIF2α phosphorylation at serine 51 and ATF4 protein levels in cells cultured in normal DMEM (Figures 4C and 4D). However, in AVIC stimulated with OGM, Gbz treatment strikingly reduced calcium deposition by 59% and decreased calcium concentration by 27% (Figure 4A), accompanied by significant decreases in calcification markers OPN and Runx2 (Figure 4B, Supplemental Figure 3A). As expected, Gbz further enhanced ISR activation in OGM-treated AVIC as revealed by higher levels of p-eIF2α, ATF4, and KDEL (Figures 4C and 4D). Additionally, Gbz improved cell survival by significantly reducing the expression of proapoptotic markers CHOP and cleaved caspase-12 (Figures 4C and 4D). Interestingly, alongside the activation of eIF2α/ATF4 signaling, Gbz treatment also increased Nox4 levels in AVIC cultured in both normal and osteogenic conditions (Figures 4C and 4D). This could be due to a potential positive feedback loop between ATF4 and Nox4.^26^

To further determine whether Gbz exerts its anticalcification effects through ATF4, endogenous ATF4 in AVIC was silenced using shATF4 adenovirus, followed by Gbz treatment. In these cells, compared with OGM stimulation alone, ATF4 knockdown increased the expression of OPN and Runx2. Notably, under these conditions, Gbz failed to suppress these calcification markers (Supplemental Figure 4A), indicating its protective effect depends on ATF4 activation. Interestingly, in Nox4-deficient AVIC, Gbz significantly reduced OPN and Runx2 levels (Supplemental Figure 4B), suggesting that although Nox4 is pivotal in regulating ATF4 signaling, ATF4 can also be activated by upstream regulators other than Nox4. Collectively, the results indicate that ATF4 plays a critical role in inhibiting osteoblast-like transdifferentiation and that the protective effect of Gbz against AVIC calcification relies on ATF4.

### Guanabenz alleviates CAVD in a rabbit model

**Figure 5:**
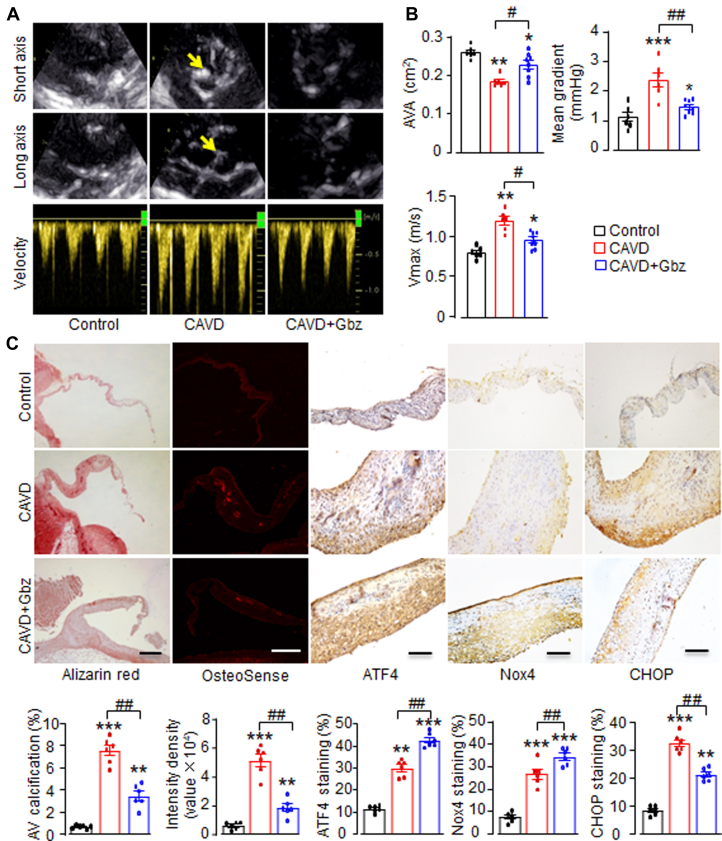
Guanabenz Alleviates CAVD in Rabbits (A) Representative echocardiographic images of the rabbit aortic valve cusps from the parasternal short-axis (top) view, long-axis (middle) view, and continuous pulsed-wave Doppler (bottom) at 18 weeks. In the control group, the aortic valves were barely visible. However, in the rabbit model of CAVD, the aortic cusps became thickened and hyperechogenic (arrow), along with increased aortic flow velocity. Treatment with Gbz (CAVD+Gbz, 1 mg/kg for 18 weeks) alleviated aortic valve stenosis. (B) Mean data of the aortic valve area (AVA), peak velocity (Vmax) and mean pressure gradients. (C) Light microscopy of the cross-sectional tissues of rabbit aortic valves with Alizarin Red stain (Scale bar: 500 μm), OsteoSense staining (scale bar: 200 μm), and immunohistochemical staining of ATF4, Nox4, and CHOP (scale bar: 100 μm). Relative quantification of positive areas shown at the bottom. n = 6-8/group. ∗*P <* 0.05, ∗∗*P <* 0.01, ∗∗∗*P <* 0.001, compared with the control cells, #*P <* 0.05, ##*P <* 0.01, compared with CAVD group, 1-way analysis of variance with a post hoc Tukey’s test. All data are mean ± SEM. Abbreviations as in Figure 3.

To further assess the therapeutic potential of Gbz against CAVD in vivo, we used a well-established rabbit model of CAVD, which closely mimics the human condition.^27^^,^^31^ In comparison to rabbits fed a normal diet, those fed a HC plus vitamin D2 (HC+vitD2) diet for 18 weeks developed clear aortic stenosis, as evaluated by echocardiography. This was demonstrated by a significant reduction in AVA, along with marked increases in transvalvular peak velocity and mean transaortic pressure gradient (Figures 5A and 5B). Remarkably, oral administration of Gbz significantly alleviated the severity of aortic stenosis. Compared with the HC+vitD2 group, Gbz treatment mitigated the decrease in AVA by 15%, and reduced the elevations in peak velocity and mean pressure gradient by 20% and 38%, respectively (Figures 5A and 5B). These results suggest that Gbz is an effective therapy for reducing aortic stenosis in rabbits in vivo.

Histological staining confirmed AV calcification in the CAVD group, revealing thickened valve leaflets with extensive calcium deposits (Figure 5C). Notably, Gbz treatment markedly reduced the calcium deposits induced by the HC+vitD2 diet (Figure 5C). Since calcification is characterized by hydroxyapatite (HAP) deposition,^4^ the fluorescence dye OsteoSense 680EX was used to assess HAP accumulation. As shown in Figure 5C, Gbz treatment significantly decreased HAP deposition compared with the untreated CAVD group, underscoring its beneficial effect against AV calcification. We also analyzed the levels of ATF4, Nox4, and apoptotic marker CHOP in AV tissues and the effects of Gbz on their expression. Immunostaining showed that Nox4 and ATF4 expression were relatively low in normal AV leaflets (Figure 5C). HC+vitD2-treated rabbits, however, exhibited significantly higher levels of Nox4 and ATF4 in diseased AVs, mirroring the up-regulation observed in human CAVD aortic leaflets (Figure 1A). Interestingly, administration of Gbz further increased the expression of both ATF4 and Nox4 in HC+vitD2-treated rabbits. Additionally, Gbz significantly reduced the expression of CHOP in calcified valves, suggesting improved cell survival (Figure 5C).

Overall, the above findings suggest that Gbz, a long-standing antihypertensive drug, shows promise as a repurposed treatment for CAVD. It exerts protective effects by enhancing AVIC survival and attenuating calcification in diseased AVs, through the activation of the eIF2α/ATF4-mediated adaptive stress response.

### Sephin 1 diminishes AVIC calcification by enhancing elF2α phosphorylation

To further support the therapeutic potential of activating adaptive stress signaling in combating CAVD, we tested the effects of Sephin1, the more specific ISR enhancer, on AVIC calcification in vitro and CAVD progression in vivo. Unlike Gbz, which binds to the α2-adrenergic receptor, Sephin1 specifically and selectively targets GADD34, thereby prolonging the benefits of adaptive p-eIF2α signaling without the adverse effects typically associated with Gbz.^23^

**Figure 6:**
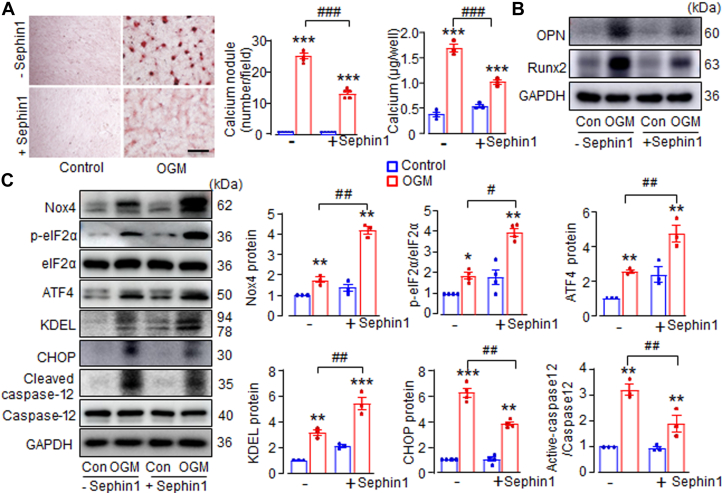
Sephin1 Diminishes AVIC Calcification Through the Increase in elF2α Phosphorylation (A) Calcium deposition by Alizarin Red staining and calcium concentration with or without Sephin1 (0.5 μmol/L, 14 days) treatment. Scale bar: 100 μm. Mean data shown at the right. (B) Immunoblots of calcification markers OPN and Runx2 with Sephin1 treatment. (C) Western blots showing the activation of p-eIF2α/ATF4/KDEL stress signaling and Nox4 protein levels, and the decreases in apoptotic markers CHOP and cleaved caspase-12 in AVIC treated with Sephin1. Mean data shown at the right. n = 3-4/group, ∗*P <* 0.05, ∗∗*P <* 0.01, ∗∗∗*P <* 0.001, compared with respective control cells (Con), #*P <* 0.05, ##*P <* 0.01, ###*P <* 0.001, compared with OGM without Sephin1 treatment. Two-way analysis of variance with a post hoc Tukey’s test. All data are mean ± SEM. Abbreviations as in Figure 1, Figure 2, Figure 3.

In AVIC treated with OGM, Sephin1 significantly reduced calcium deposition by 47% and decreased calcium concentration by 40% (Figure 6A). This was accompanied by a marked reduction in the protein levels of calcification markers OPN and Runx2 (Figure 6B, Supplemental Figure 3B), similar to the effects seen with Gbz. Consistent with its role in prolonging ISR activation, Sephin1-treated AVIC exposed to OGM exhibited elevated levels of Nox4, p-eIF2α, ATF4, and KDEL, although Sephin1 had no effect on ISR under normal culture conditions (Figure 6C). As a result of this enhanced ISR activation, Sephin1 significantly reduced the expression of the proapoptotic stress gene CHOP and improved cell survival, as demonstrated by lower levels of cleaved caspase-12 (Figure 6C).

### Sephin1 ameliorates CAVD in a mouse model

**Figure 7:**
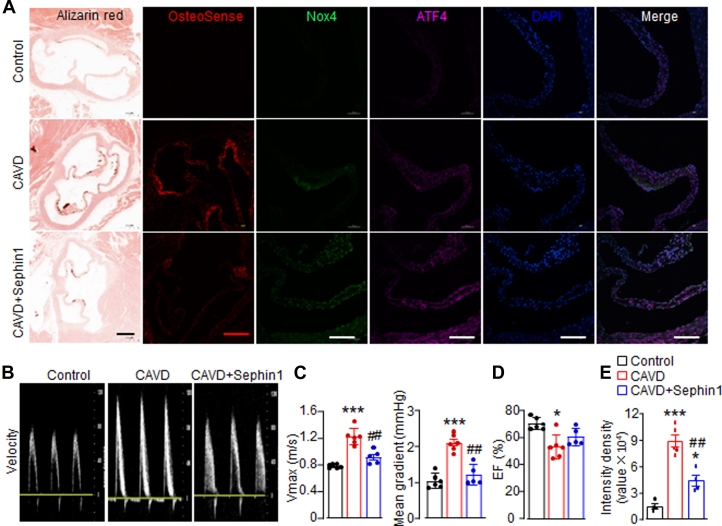
Sephin1 Ameliorates CAVD in Mice (A) Light microscopy of the cross-sectional tissues of mouse aortic valves with Alizarin Red stain (scale bar: 500 μm), OsteoSense staining (scale bar: 200 μm), and immunofluorescence staining of ATF4 and Nox4 (scale bar: 100 μm). (B) Representative echocardiographic images of pulsed-wave Doppler showing transvalvular jet velocity. (C) Mean data of peak velocity (Vmax) and the mean pressure gradient. (D) Ejection fraction (EF). (E) Relative quantification of positive OsteoSense staining. n = 5-6/group. ∗*P <* 0.05, ∗∗∗*P <* 0.001, compared with control cells, ##*P <* 0.01, compared with CAVD group, 1-way analysis of variance with a post hoc Tukey’s test. All data are mean ± SEM. Abbreviations as in Figure 5.

Finally, we evaluated the therapeutic potential of Sephin1 in mitigating valvular calcification in vivo using a mouse model of CAVD. Mice were fed an adenine diet for 3 weeks to induce renal dysfunction, followed by vitamin D injections to promote high-calcium-induced AV calcification.^29^ Alizarin Red staining revealed obvious calcified nodule formation in the CAVD group compared with the control group, which was further confirmed by OsteoSense 680EX staining (Figures 7A and 7E). Mice subjected to the adenine/Vitamin D regimen developed notable aortic stenosis, as evidenced by marked increases in transvalvular peak jet velocity and transaortic valve pressure (Figures 7B and 7C). Remarkably, intraperitoneal administration of Sephin1 led to the observed mitigation of calcified nodule formation and significant alleviation of aortic stenosis (Figures 7A to 7C). Moreover, CAVD mice exhibited impaired cardiac function (Figure 7D) and a 20% mortality rate (Supplemental Figure 5). By contrast, Sephin1-treated mice showed preserved cardiac function and reduced mortality of 10% (Figure 7D, Supplemental Figure 5), although the difference in survival did not reach statistical significance. These findings nonetheless underscore the potential beneficial effects of Sephin1 against AV calcification in vivo. Immunofluorescence staining further demonstrated elevated levels of Nox4 and ATF4 in the AVs of CAVD mice compared with control mice (Figure 7A). Interestingly, these proteins were further up-regulated in the valve tissues of CAVD mice treated with Sephin1 (Figure 7A).

In conclusion, both in vitro and in vivo experiments indicate that pharmaceutical enhancement of eIF2α/ATF4-mediated adaptive stress response by either Gbz or Sephin1 effectively mitigates AVIC and valvular calcification, offering a promising therapeutic strategy against the development of CAVD.

## Discussion

**Figure 8:**
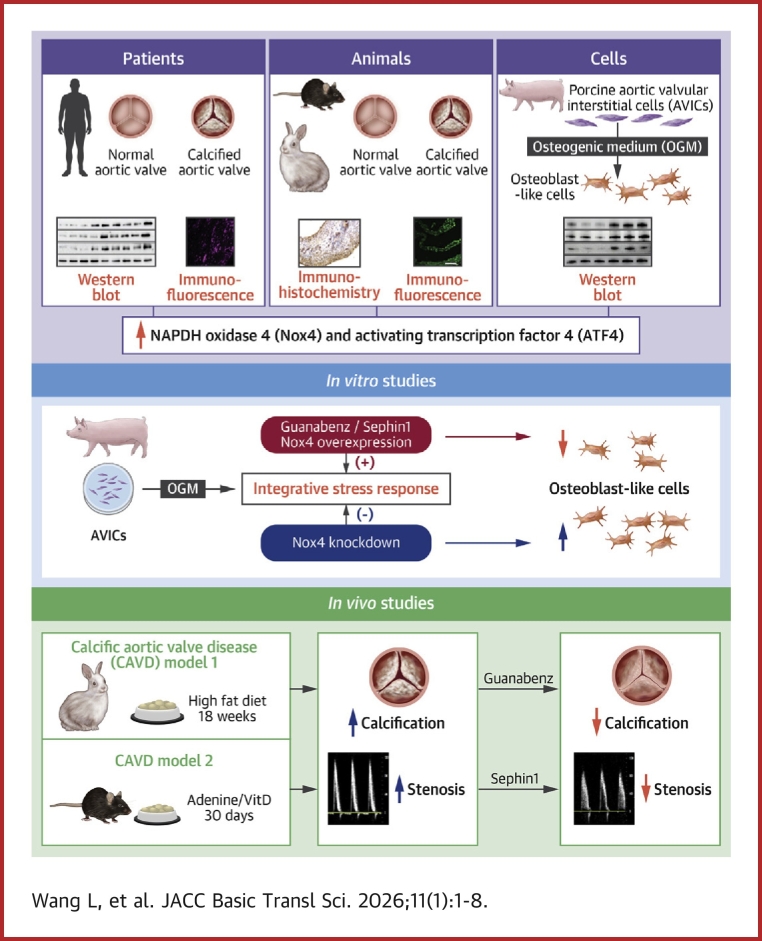
Protection Against CAVD by Pharmacological Enhancement of ISR Signaling Schematic illustrates the protection against calcific aortic valve disease (CAVD) by pharmacological enhancement of integrated stress response (ISR) signaling. The ISR effector ATF4 and the reactive oxygen species–generating enzyme Nox4 are up-regulated in the development of CAVD. A classic antihypertension drug Guanabenz. which is also a nonselective enhancer of eIF2a phosphorylation, or Sephin1, which is a selective activator of eIF2a, can effectively alleviate porcine aortic valve interstitial cell (AVIC) calcification in vitro and CAVD in vivo in rabbits and mice, respectively.

With the rising prevalence of CAVD and limited therapeutic options, the underlying mechanisms driving its initiation and progression remain unclear. In this study, we identify a concurrent increase in ISR and up-regulation of Nox4 in human calcified AVs. We further demonstrate that these changes act as compensatory adaptations to CAVD. Nox4 overexpression suppressed AVIC calcium nodule formation by enhancing ISR through the p-eIF2α/ATF4 pathway. Conversely, knockdown endogenous Nox4 promoted AVIC calcification and increased AV calcium deposits in mice. Importantly, pharmacological activation of ISR using either Gbz or Sephin1 not only mitigated AVIC osteoblastic-like differentiation in vitro, but also significantly prevented the progression of aortic stenosis in vivo in respective rabbit and mouse CAVD models. These findings highlight pharmacological enhancement of the adaptive stress response as a promising and effective strategy for protecting against CAVD (Figure 8).

It is well established that ROS play central pathophysiological roles in numerous cardiovascular diseases including CAVD,^12^ although their precise origin and contribution are not fully understood. Recent studies suggest that Nox-mediated ROS are critically involved in CAVD development; however, conflicting evidence exists regarding the specific Nox isoforms implicated.^12^ Among the 7 identified Nox homologues (Nox1 to Nox5, Duox1, and Duox2) in mammals, Nox2 and Nox4 are the most widely expressed in the heart and vasculature.^32^ Notably, Nox4 differs from Nox2 in several key aspects, including its intracellular localization, constitutive low-level activation independent of cytosolic subunits, regulation primarily by protein abundance, and its predominant production of hydrogen peroxide rather than superoxide.^33^ Our previous studies demonstrate that Nox2 levels increase in CAVD and Nox2-drived ROS contribute to exacerbated AV calcification via activation of the GSK3β/β-catenin pathway in AVIC.^27^ However, the expression profile and biological role of Nox4 in CAVD remain unclear. Some studies have reported elevated Nox4 mRNA levels in calcified AVs of CH+vitD2 rabbits,^28^ whereas others found no significant difference in Nox4 mRNA expression between normal valves and noncalcified regions of stenotic valves in humans, or even lower Nox4 mRNA levels in calcified regions of stenotic valves.^34^ This discrepancy may be attributed to differences in disease severity and patient viability. Our findings indicate that Nox4 expression is minimal in noncalcified human AVs, as shown by immunohistology. However, Nox4 protein levels are substantially increased in calcified human AVs compared with non-calcified leaflets, a result further validated by immunoblots. This up-regulation was also observed in calcified AVs of rabbits fed an HC+vitD2 diet, aligning with previous reports.^28^ The induction of Nox4 in CAVD parallels findings in the heart, where Nox4 levels are typically low under healthy conditions but increase in response to various stressors, such as hypoxia, ischemia, and hemodynamic overload.^35^ This regulation is primarily governed by transcription factors, particularly ATF4.^26^^,^^32^ Indeed, our results demonstrate that Nox4 induction coincides with elevated ATF4 levels in calcified AVs and in cultured AVIC stimulated with OGM. A substantial body of research has shown that Nox4 exerts beneficial effects in various pathophysiological settings within the heart,^35^, ^36^, ^37^, ^38^ vasculature,^39^ and other tissues.^40^ Our study provides new evidence that Nox4 plays a protective role in AVs against CAVD, at least in part through the activation of ATF4-mediated ISR in AVIC.

Cellular adaptation to various stress conditions is an intrinsic feature essential for maintaining plasticity and survival. Particularly, the ISR has emerged as a central regulatory pathway, highly conserved across all eukaryotic cells.^11^^,^^41^ The ISR networks diverse stress signals into a unified pathway initiated by the phosphorylation of eIF2α at Ser51, which suppresses global protein translation while selectively permitting the synthesis of key adaptive proteins, most notably ATF4, a hallmark indicator of ISR activation.^20^ Dysregulated ISR plays a critical role in the pathogenesis of numerous stress-related diseases,^11^^,^^20^ however, its specific role in cardiovascular pathophysiology remains largely elusive.^19^ Here, we describe that ATF4 protein levels are elevated in calcified AVs of both patients and animal models of CAVD, suggesting that ATF4-mediated ISR is adaptively activated to maintain valve homeostasis. In cultured AVIC, Nox4 overexpression increases p-eIF2α/ATF4 levels and reduces apoptotic markers, whereas Nox4 knockdown suppresses p-eIF2α/ATF4 activation and exacerbates AVIC damage. These findings indicate that ATF4 up-regulation is at least partially Nox4-dependent and that ATF4-mediated ISR enhances cell survival, reinforcing previous reports.^26^ Beyond its regulation of ATF4 and ISR activation, additional mechanisms downstream of Nox4-ATF4 signaling may contribute to cellular protection. In a mouse model of ischemic heart injury, Nox4 was shown to be essential for promoting cardiomyocyte autophagy via ATF4 activation.^42^ Furthermore, Nox4/ATF4-dependent enhancement of fatty acid oxidation has been implicated in maintaining cardiac energetic balance under chronic pressure overload.^43^ Recent studies also suggest that ATF4 induction is closely linked to nuclear factor erythroid 2-related factor 2 (Nrf2),^44^ a master transcriptional factor that is crucial for cellular antioxidant adaptation. Interestingly, Nox4 plays a pivotal role in Nrf2 activation,^37^^,^^45^^,^^46^ highlighting a potential intersection between these protective pathways. Given the complexity of Nox4-ATF4 signaling, further investigation is warranted to fully elucidate its protective mechanisms in cardiovascular diseases.

The central regulatory switch of the ISR lies in the phosphorylation of the eIF2α and its subsequent dephosphorylation by 2 phosphatase complexes, consisting of the common catalytic core PP1 and the regulatory subunit GADD34. This makes eIF2α an ideal target for modulating the ISR, with small-molecule inhibitors of these phosphatase complexes offering a valuable tool for dissecting ISR roles in disease models and presenting a promising therapeutic approach.^11^^,^^20^ Gbz, an α2-adrenergic agonist, is an oral prescription drug used to treat hypertension. Notably, Gbz has been shown to exert beneficial effects in animal models of protein misfolding diseases by inhibiting the GADD34-PP1 complex, thereby prolonging eIF2α phosphorylation.^21^^,^^22^ These encouraging findings have led to Phase I/II clinical trials evaluating Gbz’s dosage and safety in patients with multiple sclerosis.^16^^,^^41^ In this study, we found that p-eIF2α/ATF4 levels are low in normal AVIC but can be activated by osteogenic medium. Treatment with Gbz further enhanced ISR activation, decreased apoptotic markers, and significantly reduced calcium nodule formation by 59%—an effect that was dependent on the presence of ATF4. To assess Gbz’s therapeutic potential in vivo, we used an established HC-vitD2-induced rabbit CAVD model.^27^ Compared with the HC-vitD2 group, oral administration of Gbz for 18 weeks significantly increased ATF4 and Nox4 levels, reduced CHOP expression, mitigated calcium deposition as shown by Alizarin Red and OsteoSense staining, alleviated aortic stenosis as evidenced by decreased transvalvular peak and mean pressure gradient on echocardiography, and mitigated cardiac dysfunction. To the best of our knowledge, this study is the first to identify Gbz as a potential repurposed treatment for CAVD.

Given Gbz’s effects on α2-adrenergic receptors and its potential anti-inflammatory properties,^47^ we further examined sephin1, a Gbz derivative that selectively binds to GADD34, sustaining eIF2α phosphorylation only under stress conditions,^23^ although precise mechanism remains under active investigation.^48^ Sephin1 demonstrated a similar effect to Gbz, showing no basal impact on the p-eIF2α/ATF4 pathway but significantly enhancing ISR activation in AVIC under osteogenic stimulation, leading to a 47% reduction in calcium deposition. Furthermore, intraperitoneal administration of sephin1 markedly reduced calcified nodule formation and alleviated aortic stenosis in a mouse model of CAVD induced by acute renal dysfunction. These observations suggest that maintaining p-eIF2α levels within a narrow physiological range is crucial for normal AVIC function, but the ISR needs to adaptively switch on during valve calcification. Adjusting the extent of ISR activation through compound-based strategies to enhance p-eIF2α levels presents a novel therapeutic approach for managing CAVD.

### Study Limitations

First, although the ISR plays a crucial role in normal physiology, its activation leads to remarkably complex consequences. Dysregulation of the ISR manifests in diverse pathologies, selectively affecting specific tissues and cell types. It was reported that global or smooth muscle cell-specific deletion of ATF4 could alleviate vascular calcification.^49^ Although the mechanisms underlying CAVD and vascular calcification are distinct, the varying findings regarding ISR’s role in disease onset and progression may stem from differences in animal models, transgenic lines, and the timing or extent of ISR modulation during disease development.^20^ Additionally, the contribution of Nox4 is well recognized as being highly cell-type specific and context-dependent.^32^ Therefore, employing AVIC-specific Nox4 and ATF4 gene-modified mice would provide valuable insights. Second, compared with the clinical scenario of advanced aortic stenosis, the development of CAVD in the animal models used in this study occurs relatively quickly, with less severe stenosis. Further research is needed to assess potential off-target effects of Gbz and Sephin1, particularly if long-term or high-dose administration is required. Caution is also warranted when extrapolating these findings to clinical applications. Third, since AV area and transvalvular velocity are influenced by cardiac function and afterload—and given that Gbz is a known α2-adrenergic agonist capable of modulating blood pressure in rabbits^50^—further investigation using invasive hemodynamic assessments in animal CAVD models is necessary. Fourth, emerging evidence indicates sex difference in CAVD patients,^51^ thus future studies should include both male and female animals to ensure comprehensive analysis.

## Conclusions

This study reveals that the ROS-generating enzyme Nox4, along with ATF4—a key marker of ISR activation, are up-regulated in calcified AVs. Both Gbz and Sephin1, inhibitors of the GADD34-PP1 complex that prolong eIF2α phosphorylation, effectively attenuate the osteoblastic-like differentiation of AVIC in vitro, and alleviate AV calcification and stenosis in animal models in vivo via promoting p-eIF2α/ATF4 pathway in AVIC. These findings imply that pharmacological enhancement of the adaptive stress response offers protection in CAVD. Notably, the classic drug Gbz holds potential for repurposing as an early intervention strategy to mitigate this debilitating condition.

Perspectives **COMPETENCY IN MEDICAL KNOWLEDGE:** The ISR, an evolutionarily conserved signaling network essential for maintaining cellular health, is adaptively activated during the progression of CAVD. Pharmacologically enhancing the ISR, such as with the repurposed antihypertensive drug Gbz, holds promise as a potential strategy to improve disease outcomes.

**TRANSLATIONAL OUTLOOK:** Further studies are needed to evaluate the therapeutic effectiveness of Gbz and Sephin1 in established CAVD, as well as to explore the potential of other ISR activators.

### Data Availability

Data can be made available upon reasonable request from the corresponding author.

## Funding Support and Author Disclosures

This study was supported by the National Natural Science Foundation of China (32171179), Key Research Project of the Heart Center of Xinxiang Medical University (2017360), and grants from British Heart Foundation (PG/22/11055 to Dr Zhang, CH/1999001/11735 to Dr Shah). The authors have reported that they have no relationships relevant to the contents of this paper to disclose.
