# Supplementary material for: Pharmacological Enhancement of Integrated Stress Response Confers Protection in Calcific Aortic Valve Disease
Source: JACC Basic Transl Sci. 2025 Dec 15;11(1):101433. doi: 10.1016/j.jacbts.2025.101433 (PMC12769411; doi:10.1016/j.jacbts.2025.101433)
Supplement: Supplemental Figures 1-5 [file mmc1.docx]

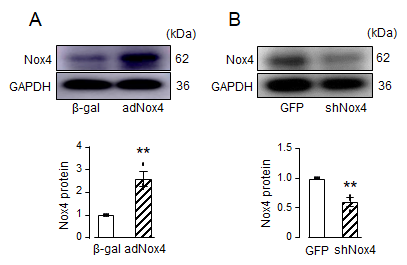


**Supplemental Figure 1.** Representative immunoblots (upper) and relative quantification (lower) for Nox4 in AVIC after adenovirus transfection to overexpress (A) or knockdown (B) Nox4. AVIC transfected with β-galactosidase (β-gal) virus or green fluorescent protein (GFP) virus as respective controls. n=3/group. **p<0.01. Student’s unpaired t-test. All data are mean ± SEM.


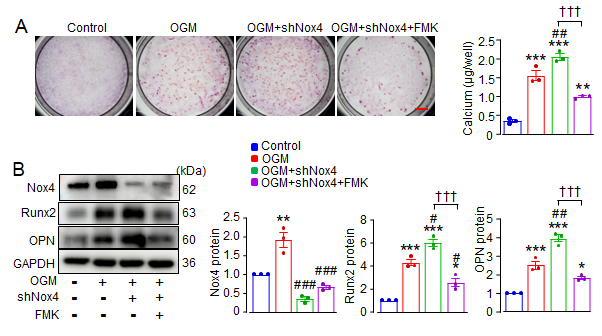


**Supplemental Figure 2.** Inhibition of apoptosis with pan-caspase inhibitor FMK attenuates calcification in Nox4-deficient AVIC. (A) Calcium deposition assessed by Alizarin Red staining. Scale bar: 2 mm. Quantification of calcium concentration is shown on the right. n=3/group. (B) Immunoblots (left) and corresponding quantification (right) of osteopontin (OPN) and runt-related transcription factor 2 (Runx2) in AVIC. n=3/group. *p<0.05, **p<0.01, ***p<0.001, compared with control groups (Con), #p<0.05, ##p<0.01, ###p<0.001, compared with OGM groups, †††p<0.001, compared with OGM+shNox4 groups. 2-way analysis of variance with a post hoc Tukey’s test. All data are mean ± SEM.


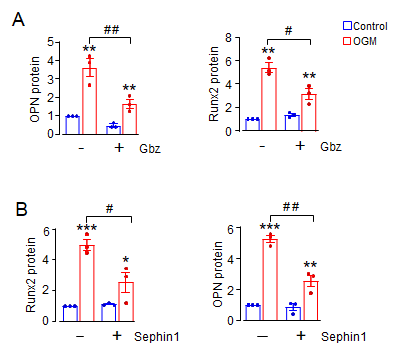


**Supplemental Figure 3.** Mean data of protein immunoblots of calcification markers OPN and Runx2 in AVIC with or without treatment of Guanabenz (Gbz) (A) or Sephin1 (B). n=3/group, *p<0.05, **p<0.01, ***p<0.001, compared with respective controls, #p<0.05, ##p<0.01, compared with calcification without Gbz or Sephin1 treatment. 2-way analysis of variance with a post hoc Tukey’s test. All data are mean ± SEM. Abbreviations as in Supplemental Figure 2.


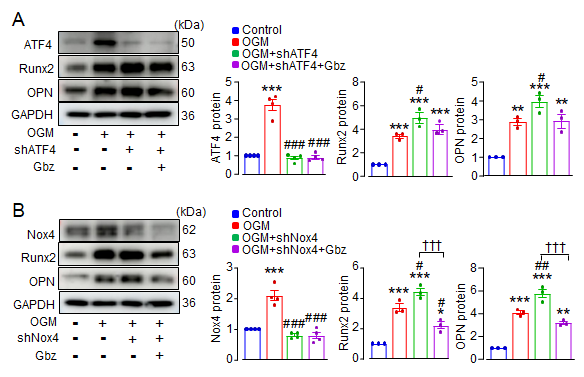


**Supplemental Figure 4.** The protective effect of Guanabenz (Gbz) against AVIC calcification is dependent on ATF4. (A) Effect of ATF4 knockdown using shATF4 adenovirus, and (B) effect of Nox4 knockdown using shNox4 adenovirus, on protein levels of osteopontin (OPN) and runt-related transcription factor 2 (Runx2) in AVIC with or without Gbz treatment. Mean data shown at the right. n=3/group. *p<0.05, **p<0.01, ***p<0.001, compared with control groups, #p<0.05, ##p<0.01, ###p<0.001, compared with OGM groups, †††p<0.001, compared with OGM+shNox4 or OGM+shATF4 groups. 2-way analysis of variance with a post hoc Tukey’s test. All data are mean ± SEM.


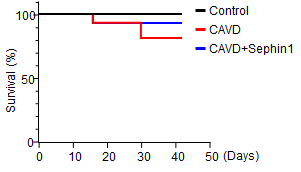


**Supplemental Figure 5.** Kaplan–Meier survival analysis of normal control mice, CAVD mice, and CAVD mice treated with 1 mg/kg Sephin1. n=10-15 mice/group.
