# Supplementary material for: Pharmacological Enhancement of Integrated Stress Response Confers Protection in Calcific Aortic Valve Disease
Source: JACC Basic Transl Sci. 2025 Dec 15;11(1):101433. doi: 10.1016/j.jacbts.2025.101433 (PMC12769411; doi:10.1016/j.jacbts.2025.101433)

Representative figure

Figure 1B

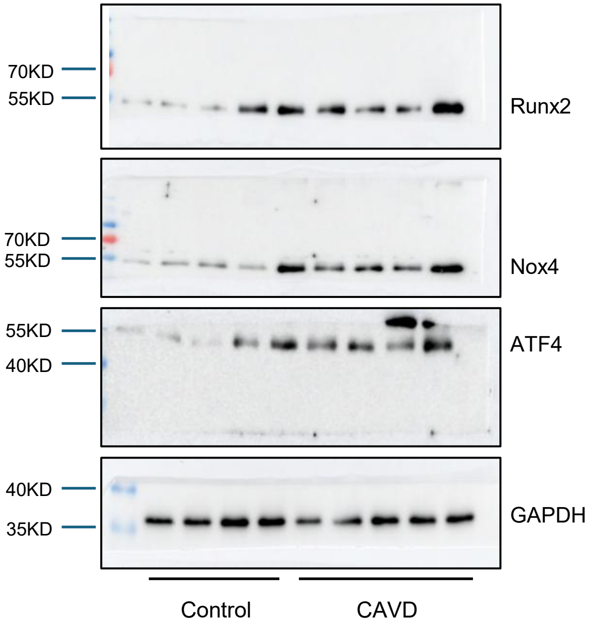

Representative figure

Figure 1C

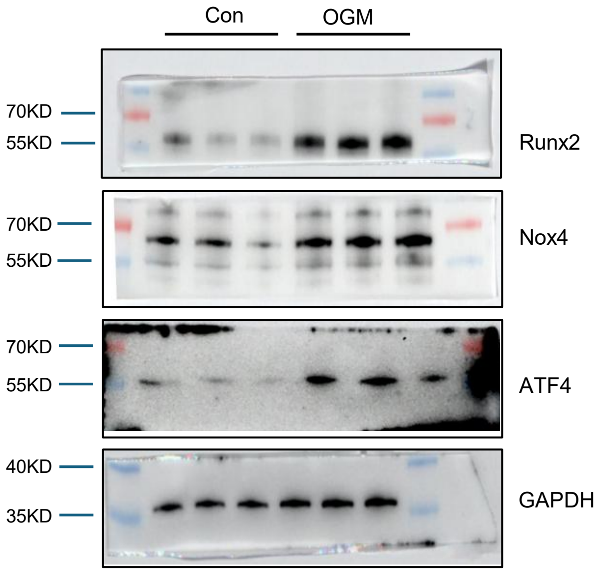

Representative figure

Figure 2C

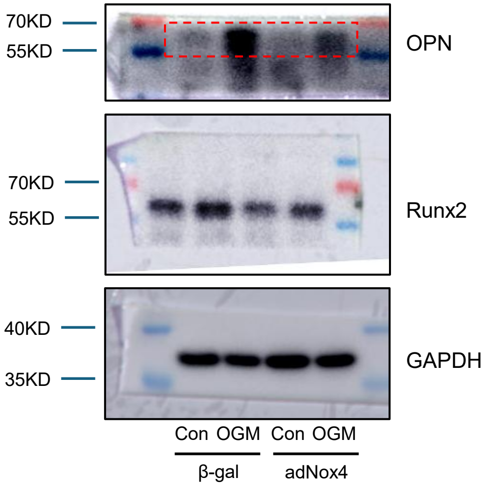

Representative figure

Figure 2D

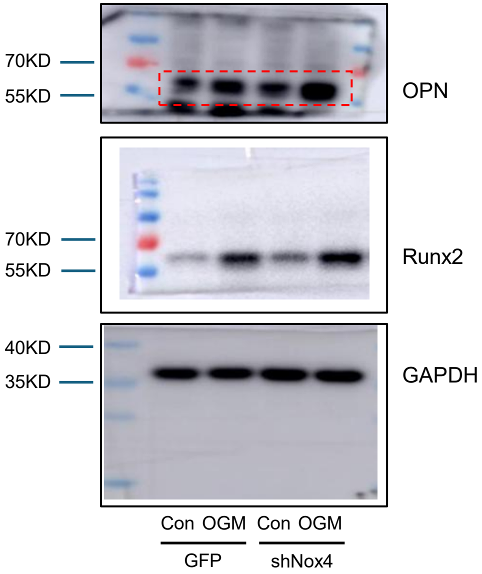

Representative figure

Figure 3A

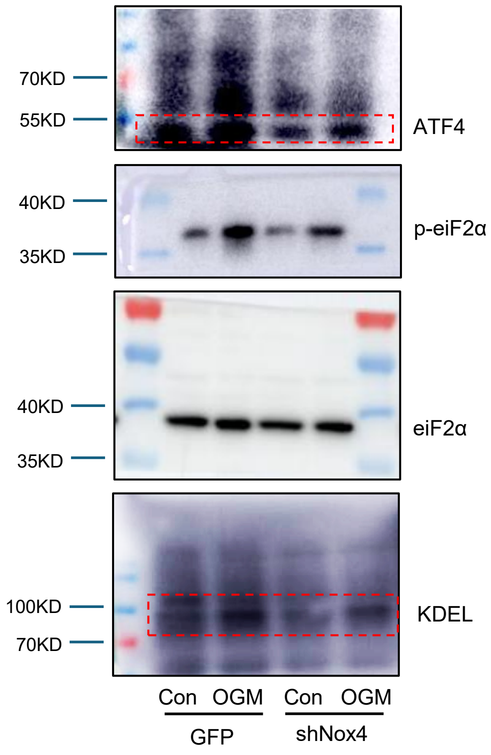

Representative figure

Figure 3A

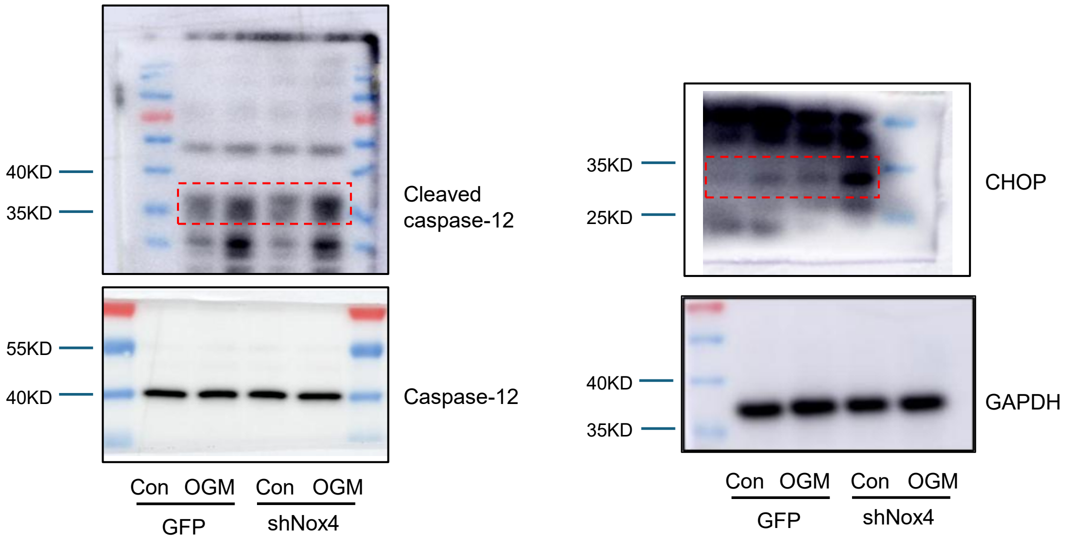

Representative figure

Figure 3B

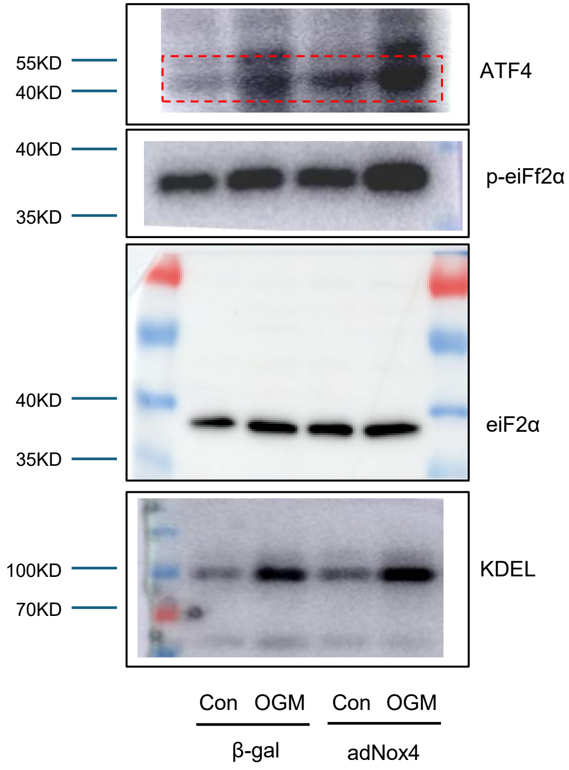

Representative figure

Figure 3B

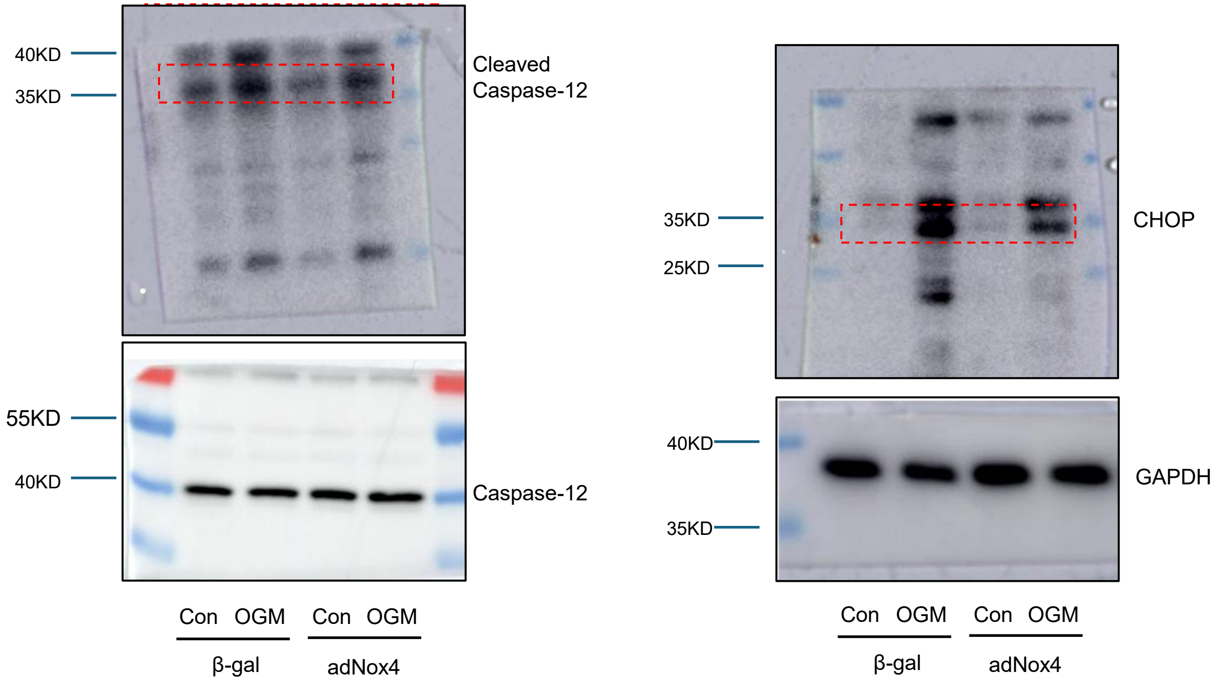

Representative figure

Figure 4B

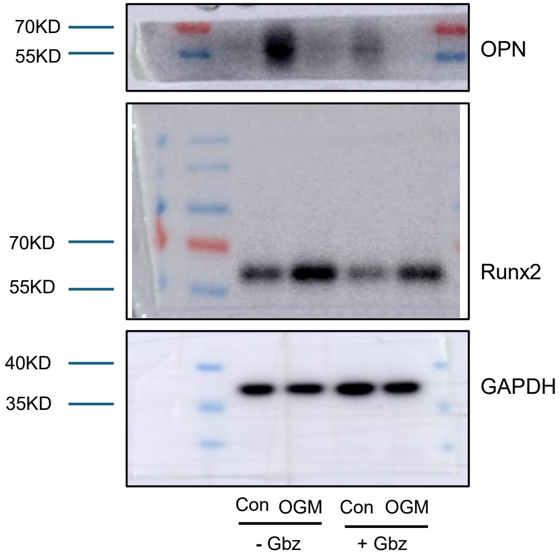

Representative figure

Figure 4C

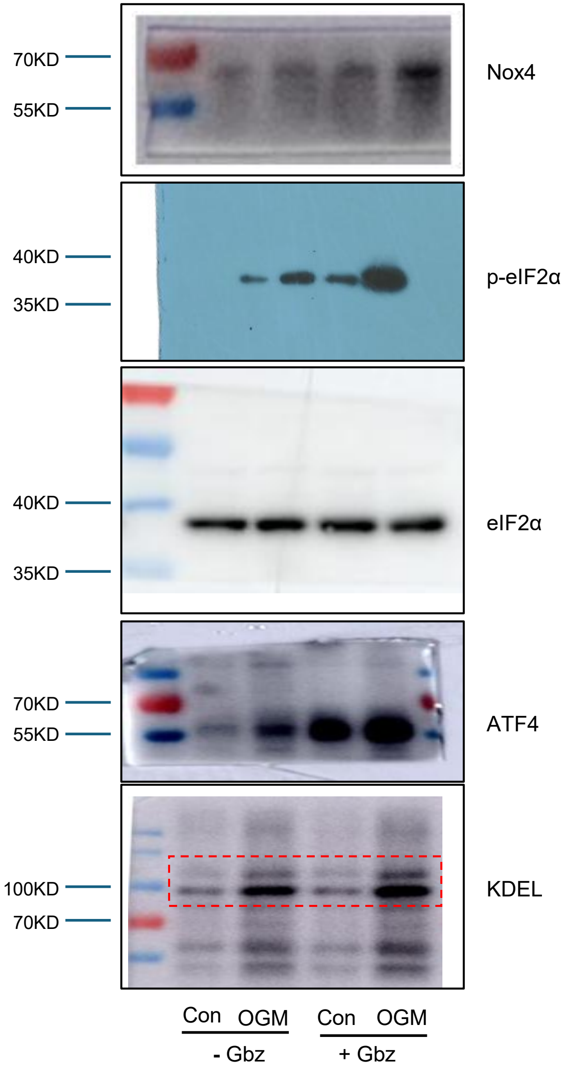

Representative figure

Figure 4C

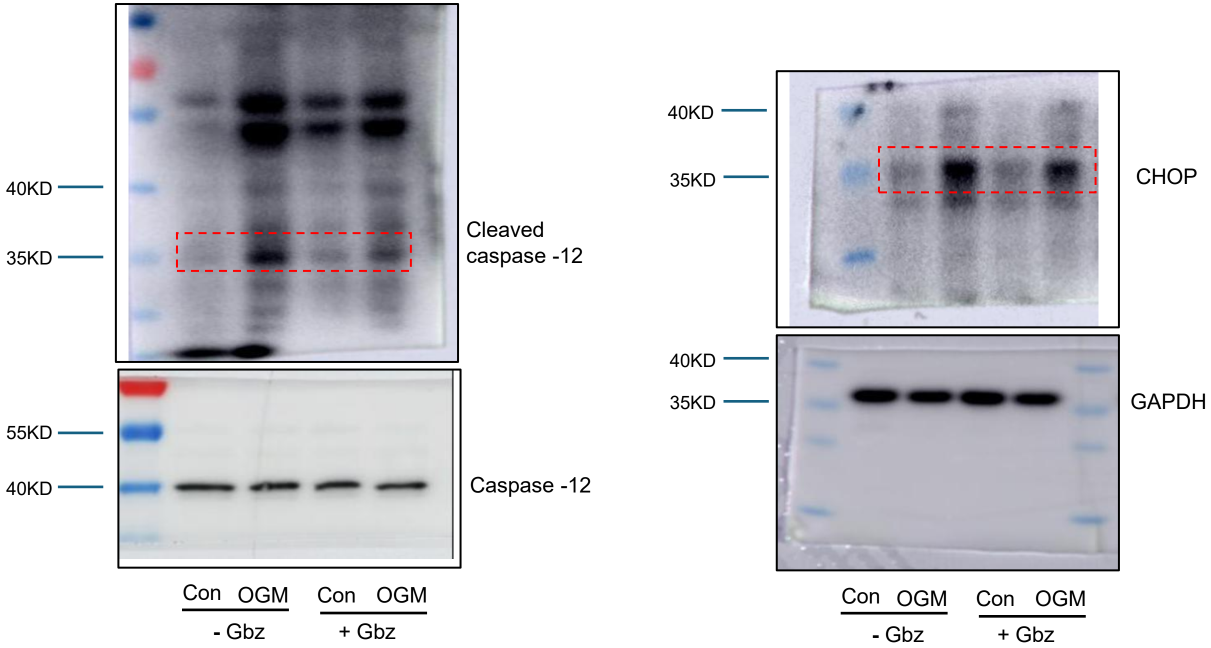

Representative figure

Figure 6B

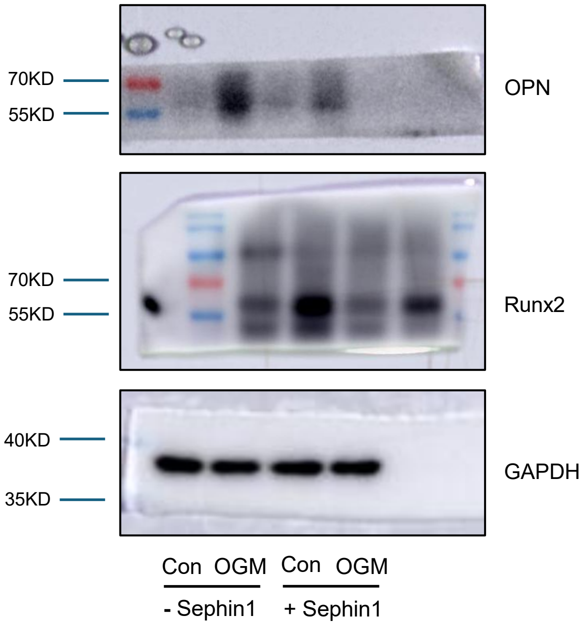

Representative figure

Figure 6B

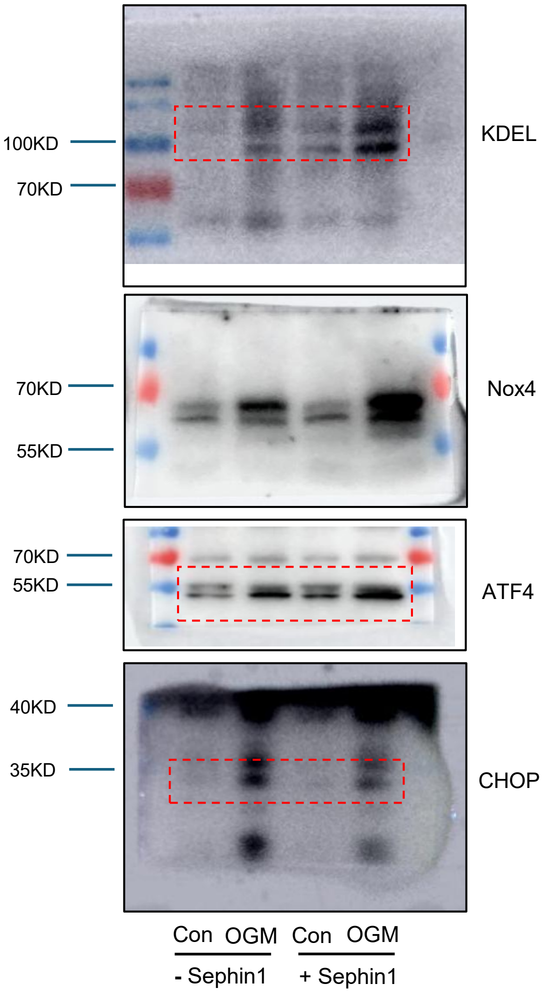

Representative figure

Figure 6B

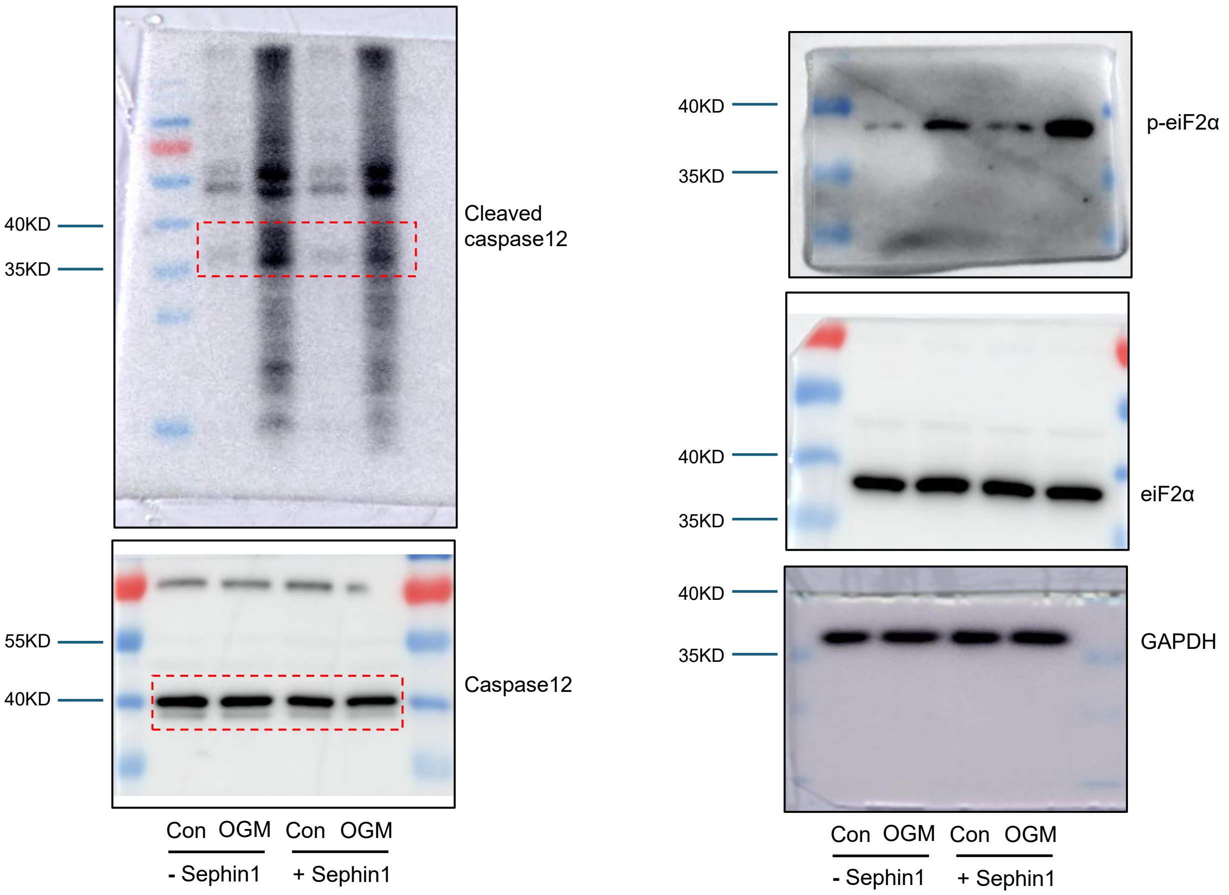

Representative figure

Supplementary Figure 1A and 1B

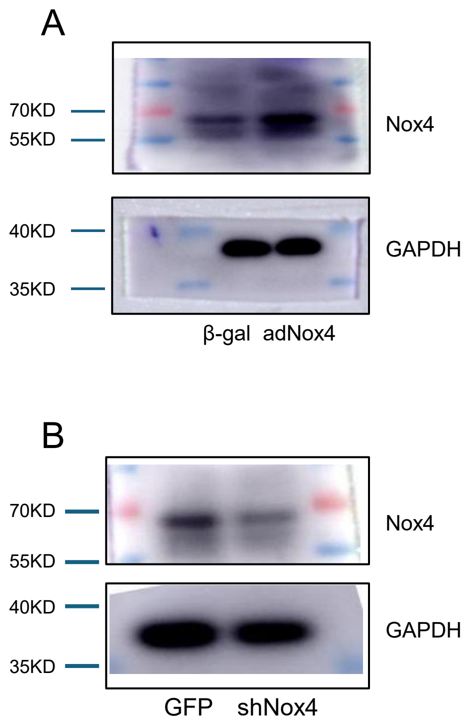

Representative figure

Supplementary Figure 2B

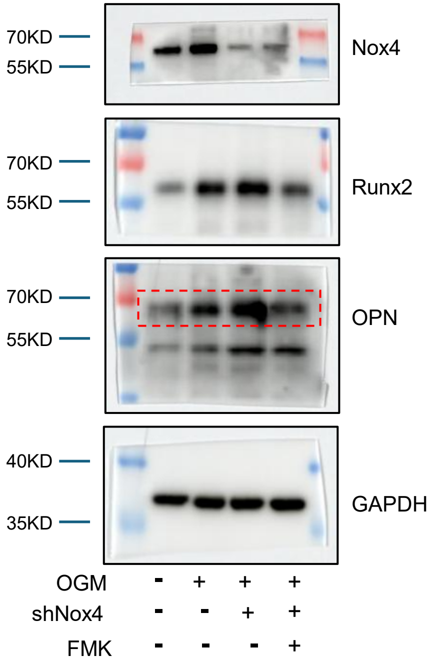

Representative figure

Supplementary Figure 4A

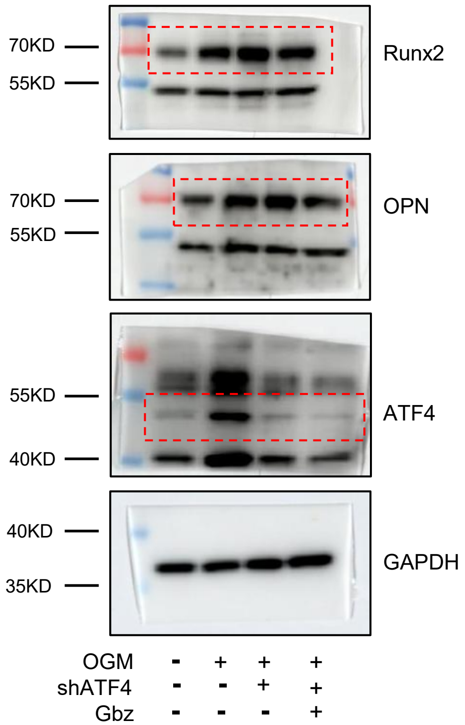

Representative figure

Supplementary Figure 4B

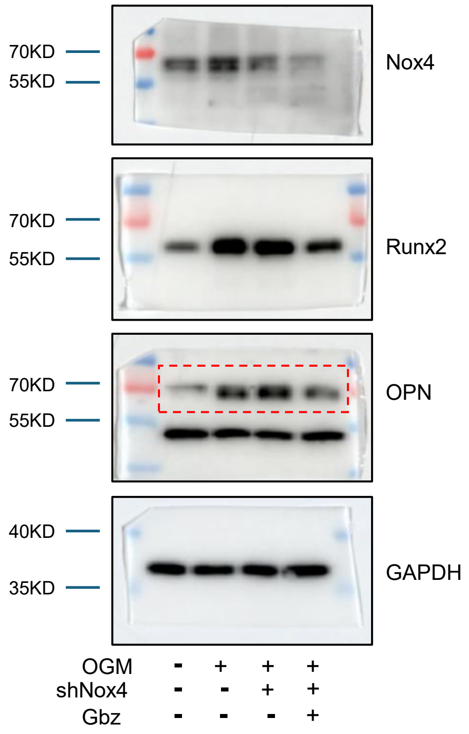

Supplement: Supplemental Figures 1-6 and Supplementary Figures 1-4 [file mmc2.pdf]
